# A novel mutation and first report of dilated cardiomyopathy in ALG6-CDG (CDG-Ic): a case report

**DOI:** 10.1186/1750-1172-5-7

**Published:** 2010-04-16

**Authors:** Mohammed Al-Owain, Sarar Mohamed, Namik Kaya, Ahmad Zagal, Gert Matthijs, Jaak Jaeken

**Affiliations:** 1 Department of Medical Genetics, King Faisal Specialist Hospital and Research Centre, Riyadh, Saudi Arabia; 2 College of Medicine, Alfaisal University, Riyadh, Saudi Arabia; 3 Department of Pediatrics, Saad Hospital, Al-Khobar, Saudi Arabia; 4 Department of Genetics, King Faisal Specialist Hospital and Research Centre, Riyadh, Saudi Arabia; 5 Center for Human Genetics, University Hospital Gasthuisberg, Leuven, Belgium; 6 Center for Metabolic Disease, University Hospital Gasthuisberg, Leuven, Belgium

## Abstract

Congenital disorders of glycosylation (CDG) are an expanding group of inherited metabolic diseases with multisystem involvement. ALG6-CDG (CDGIc) is an endoplasmatic reticulum defect in N-glycan assembly. It is usually milder than PMM2-CDG (CDG-Ia) and so is its natural course. It is characterized by psychomotor retardation, seizures, ataxia, and hypotonia. In contrast to PMM2-CDG (CDGIa), there is no cerebellar hypoplasia. Cardiomyopathy has been reported in a few CDG types and in a number of patients with unexplained CDG. We report an 11 year old Saudi boy with severe psychomotor retardation, seizures, strabismus, inverted nipples, dilated cardiomyopathy, and a type 1 pattern of serum transferrin isoelectrofocusing. Phosphomannomutase and phosphomannose isomerase activities were normal in fibroblasts. Full gene sequencing of the ALG6 gene revealed a novel mutation namely c.482A>G (p.Y161C) and heterozygosity in the parents. This report highlights the importance to consider CDG in the differential diagnosis of unexplained cardiomyopathy.

## Introduction

Inborn errors of metabolism (IEM) account for only 5% of all pediatric cardiomyopathy and 15% of patients with known causes. More than 40 different IEM involving cardiomyopathy exist, including energetic diseases with fatty acid oxidation defects and mitochondrial respiratory chain defects, organic acidurias, glycogen storage diseases, lysosomal storage disorders and congenital disorders of glycosylation [1]. Cardiomyopathy has been reported in PMM2-CDG (The novel CDG nomenclature is used ie the non-italicized gene symbol followed by: -CDG [2,3]), ALG12-CDG (CDG-Ig), DK1-CDG (CDG-Im) and COG7-CDG (CDG-IIe), as well as in patients with an unexplained CDG. Both hypertrophic and dilated cardiomyopathies have been described in CDG, with no common pattern observed in a particular CDG [4].

Congenital disorders of glycosylation (CDG) are a rapidly growing group of inherited metabolic disorders due to defects in the synthesis of glycans and their attachment to proteins and lipids [5]. They show a broad range of clinical manifestations and may be highly variable within the same subtype and even among affected siblings [6,7]. The method of choice for screening of these disorders is still isoelectrofocusing of serum transferrins (IEF) [8]. The N-glycosylation defects can be divided in two groups: CDG-I caused by dysfunction of glycan assembly, and CDG-II, caused by abnormal glycan processing [9,10]. CDG-I patients usually show a type 1 serum transferrin IEF pattern, and CDG-II patients a type 2 pattern.

ALG6-CDG (MIM #603147) is caused by defects in the *ALG6 *gene coding for Dol-P-Glc:Man9-GlcNAc2-P-P-Dol glucosyltransferase (glucosyltransferase 1). It is as a rule milder than PMM2-CDG (CDGIa) and is characterized by psychomotor retardation, axial hypotonia, seizures, ataxia, strabismus, feeding difficulties and a very low serum cholesterol and clotting factor XI [11-14]. Other reported features include retinal degeneration [10], deep vein thrombosis, and pseudotumor cerebri [15].

Here we report a Saudi child with ALG6-CDG and dilated cardiomyopathy caused by a novel mutation.

## Patient Report

**Figure 1:**
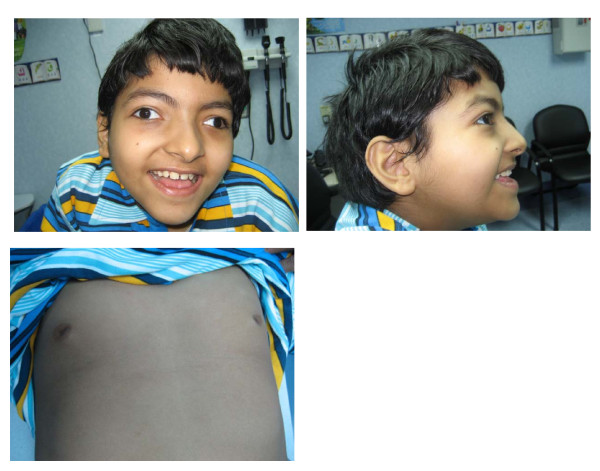
**The patient at the age of 9 years**. Note the low hairline, broad nasal bridge, hypetelorism, bilateral strabismus, large ears, wide mouth, thin upper lip, and widely spaced inverted nipples.

A 9 year old Saudi boy (Fig. 1) was referred for evaluation of psychomotor retardation, hypotonia and dilated cardiomyopathy. He was born at 40 weeks gestation after a normal pregnancy and delivery with a birth weight of 4.6 kg. Hypotonia without feeding problems was noted in the neonatal period, bilateral alternating squint at two months of age, and hypokinesia at four months of age. At one year of age, he developed febrile seizures followed by afebrile partial epilepsy that responded well to carbamazepine. At three years of age, he presented with recurrent episodes of difficulty breathing and fatigability. Chest X-ray revealed cardiac enlargement with increased pulmonary vascularity. Echocardiography showed moderate dilatation and dysfunction of the left ventricle (LV). The end-systolic LV dimension was 3 (1.7-2.5 cm) corresponding to a Z-score of 4.3, while the end-diastolic LV dimension was 4.2 (2.9-3.9 cm) and the Z-score was 3.4. The ejection fraction and ejection fraction shortening were slightly subnormal at 56% and 27%, respectively. The interventricular septum thickness was normal. There was no mitral regurgitation and no pericardial effusion. These findings confirmed moderate cardiomyopathy of the dilated type. He was subsequently placed on captopril at a dose of 6.25 mg three times daily that was continued for five years. Captopril was just recently weaned off with stabilization of the cardiac function. At the age of 6 years, he was not able to sit unsupported, was nonverbal and is completely dependent on the family for care. On physical examination at the age of 7 years, the child was wheelchair bound with severe mental retardation and no speech. His head circumference and weight were on the 50 centile. His height was on the 25 centile. He had brachycephaly, bilateral esotropia, coarse hair with double hair whorl, low anterior hair line, broad nasal bridge, widely spaced eyes, prominent large ears, short philtrum, wide mouth with a thin upper lip, small teeth, widely spaced inverted nipples, bilateral cryptorchidism, reduced muscle bulk and tone with axial hypotonia. Deep tendon reflex were difficult to elicit. Finger joints were hyperextensible but knees and hips showed limited joint extension. Routine laboratory investigations showed normal urinalysis, normal complete blood count, blood glucose, thyroid and kidney function tests, serum amino acids, lactate, acylcarnitine profile, and urine organic acids. Serum GOT was 37 (17-59 U/L), GPT 24 (21-72 U/L). Cholesterol and clotting factor XI were not available. CSF examination was normal for cells, protein, glucose and lactate. Brain MRI revealed widening of CSF spaces and ventricular system with normal brain stem and cerebellum. EMG, nerve conduction velocity, funduscopy, hearing assessment and abdominal ultrasound were normal. Serum transferrin IEF showed a type 1 pattern. Phosphomannomutase 2, and phosphomannose isomerase activities were normal in fibroblasts. Molecular genetic testing diagnosed ALG6-CDG with a novel mutation namely c.482A>G (p.Y161C).

The family history revealed that the patient was the fourth child born to healthy consanguineous parents, and he had two healthy siblings. His elder sister died at 7 years of age with a similar but more severe syndrome comprising microcephaly, deafness, blindness, severe psychomotor retardation and intractable seizures. She had no cardiac symptoms. She was investigated thoroughly for metabolic disorders including mitochondrial disorders and chromosomal abnormalities; however, no diagnosis was made at that time.

## Discussion

ALG6-CDG is the second most frequently described N-glycosylation defect besides PMM2-CDG. At least 30 patients have been diagnosed [16]. Twenty one different mutations are listed in the Human Genome Mutation database http://www.hgmd.cf.ac.uk: sixteen point mutations, four deletions (including a large deletion enclosing the complete ALG6 gene), and one insertion. The c.998C>T (p.Ala333Val) mutation accounts for the majority of the alleles. The present patient is homozygous for a previously not reported mutation. The mutation found in this study is highly likely to be pathogenic for the following reasons a) PANTHER[17], POLYPHEN[18], and SIFT[19] bioinformatics tools all predicted that the change be deleterious and probably damaging, b) tyrosine in position p. 161 is phylogenetically conserved. His clinical presentation comprises the known features of ALG6-CDG: axial hypotonia, moderate to severe psychomotor retardation, strabismus, epilepsy, without hepatomegaly, proteinuria, retinopathy and cerebellar hypoplasia. However, in addition, he shows dilated cardiomyopathy, a feature previously not reported in ALG6-CDG. The severity of the dilated cardiomyopathy in the present patient was moderate and required long-term cardiac treatment with captopril.

Cardiomyopathy (hypertrophic and dilated) has been reported in PMM2-CDG [20-25], in ALG12-CDG [26], in DK1-CDG [27], and in COG7-CDG [28], as well as in a number of patients with an unexplained CDG (CDG-Ix and CDG-IIx) [4,21,29-33]. The age of diagnosis of cardiomyopathy in reported cases ranged from the first week of life to 7 years of age, and in a few cases it was detected prenatally [2,25,31]. In addition, this is the first report of CDG from Saudi Arabia and it is likely that this condition is underdiagnosed in this region often due to poor access to appropriate metabolic and genetic testing.

In conclusion, we present the first case of ALG6-CDG associated with mild dilated cardiomyopathy due to a novel mutation in the *ALG-6 *gene. We feel from this report and along with previous reports that patients with unexplained (particularly syndromatic) cardiomyopathy should be investigated for CDG.

## Abbreviations

CDG: congenital disorder(s) of glycosylation; IEF: isoelectrofocusing; IEM: Inborn errors of metabolism.

## Consent

Written informed consent was obtained from the patient's parents for publication of this case report and accompanying images. A copy of the written consent is available for review by the Editor-in-Chief of this journal.

## Competing interests

The authors declare that they have no competing interests.

## Authors' contributions

MA, SM, AZ and JJ were involved in the clinical evaluation and follow-up of the patient, the data analysis and interpretation, and drafted the manuscript. GM carried out the molecular genetic studies and the interpretation of the results. NK assisted in performing the bioinformatics analysis and was involved in the write-up of the manuscript. All authors read and approved the final manuscript.
